# AI-enhanced virtual reality martial arts training: how technology readiness, instructional design, usefulness, and instructor competency drive learning performance through cognitive absorption

**DOI:** 10.1038/s41598-026-35749-2

**Published:** 2026-01-27

**Authors:** Zhong Xing Zhang

**Affiliations:** College of Humanities and Medicine, Anhui Medical University, Hefei, 230032 China

**Keywords:** AI-enhanced virtual reality, Cognitive absorption, Technology readiness, Instructional design quality, Structural equation modeling, Education, Information systems and information technology, Psychology, Psychology, Science, technology and society

## Abstract

Artificial intelligence–enhanced virtual reality (AI-VR) holds significant potential for complex psychomotor skills training, such as in martial arts. However, the psychological pathways through which its features influence learning outcomes are not well understood. This study introduces and tests the Technology-Enhanced Experiential Learning (TEEL) framework, which posits cognitive absorption as the central mediator between key factors and learning performance. A mixed-methods design was employed. Quantitatively, survey and system-usage data were collected from 847 martial artists across 23 facilities after six supervised AI-VR sessions. Structural equation modeling (SEM) and bootstrap mediation analyses were used to test the framework. Qualitatively, 45 semi-structured interviews were thematically analyzed and integrated with the quantitative findings. The model demonstrated strong fit and explanatory power (R² = 0.732 for Learning Performance). Technology Readiness, Instructional Design Quality, Instructor Competency, and Perceived Usefulness all significantly predicted Cognitive Absorption (β = 0.387 to 0.251, p < 0.001), which in turn strongly predicted Learning Performance (β = 0.791, p < 0.001). Cognitive Absorption fully mediated all antecedent relationships. The AI-enhanced model outperformed a VR-only baseline, and usage analytics confirmed significant skill improvement, with strike accuracy increasing from 64.2% to 87.6%. The findings validate the TEEL framework, establishing cognitive absorption as the core mechanism through which technological and pedagogical factors enhance learning in AI-VR martial arts training. This underscores the value of AI components and highlights design priorities for creating deeply engaging and effective training systems.

## Introduction

AI–VR integration is transforming sports training. It is especially well-suited to martial arts, where precise movement analysis, instant feedback, training safety, and consistent instruction across skill levels and locations are paramount. Building on technology-enhanced learning, cognitive psychology, HCI, and educational technology, foreground cognitive absorption—focused attention, temporal dissociation, and heightened interactivity—as a key mechanism linking technological features (visual quality, interactivity, sound quality) to learning outcomes in immersive settings. While promising, this mechanism remains under-examined for AI-enhanced VR in complex psychomotor domains such as martial arts, despite growing empirical signals of feasibility and benefit.

Evidence specific to martial arts and combat sports shows rapid progress. Immersive reviews and systems point to rising potential for traditional training^1, 2^, and empirical studies report enhanced kumite response behavior and transfer to performance^3, 4^. Methodological advances validate the reliability of VR-based reaction-time measurement in fighters^5, 6^, while rehabilitation and junior performance gains are demonstrated through hybrid mixed reality and targeted VR protocols^7, 8^. Work on autonomous VR karate characters extends training and research possibilities^9^. Acceptance and UX are critical determinants: the uptake of head-mounted displays by athletes has been investigated^10^, and display modality (3D-360 ° VR vs. 2D) measurably shapes gaze, head excursion, and cognitive workload in boxing-specific anticipation^11^.

Beyond martial arts, studies across sports substantiate VR’s role in complex motor learning and perceptual-cognitive development. Investigations span gaze behavior and motor-skill learning processes^12, 13^, advantages over video-screen stimulation in youth football^14^, temporal discrimination in softball batting^15^, and multi-scale analyses of basketball throwing^16^. Transfer from VR to real-world performance is repeatedly observed, with effects varying by dose and context^17, 18^, and conventional instruction is evidenced in learning gymnastics elements^19^. Syntheses consolidate the field^20, 21, 22^; Faure and colleagues^23^. At the same time, advanced implementations illustrate gaze-contingent boxing, real-time visual feedback in golf, and age-sensitive body visualization effects^24, 25, 26^.

Important gaps remain: many studies isolate single features or outcomes, under-specify psychological mediators, and do not fully explain how technology readiness, instructional design quality, perceived usefulness, and instructor competence jointly drive participation and effectiveness in AI-VR environments. To address this, propose and test a Technology-Enhanced Experiential Learning (TEEL) framework that integrates technology acceptance, educational psychology, and HCI, with cognitive absorption mediating the relationships among technological/pedagogical factors and learning performance. Using a mixed-methods structural equation model with qualitative triangulation across 847 martial artists at 23 training locations, find support for all hypotheses, extend the evidence on technology-enhanced learning, and derive practical guidance for next-generation AI-VR training systems optimized around Technology Readiness Support, Instructional Design Quality, Virtual Instructor Competency, and Perceived System Usefulness.

## Method

### Technology-enhanced experiential learning (TEEL) framework

The Technology-Enhanced Experiential Learning (TEEL) framework synthesizes cognitive psychology, technology acceptance, and educational technology to explain the effectiveness of learning in AI-enhanced VR. It models performance as an outcome of interacting technological features, pedagogical design, individual technology readiness, and psychological mediators, with cognitive absorption posited as the central mechanism translating external affordances and instructional inputs into meaningful learning. TEEL draws on the Technology Acceptance Model (linking perceived usefulness to engagement and outcomes), the Technology Readiness Index (capturing predispositions toward adoption and effective use), flow theory and cognitive-absorption research (deep, goal-directed engagement), instructional design theory (quality of learning supports), and instructor-competency scholarship (quality of guidance in mediated settings). Advancing prior models, TEEL explicitly incorporates AI capabilities, adaptive difficulty control, intelligent motion capture for biomechanical feedback, natural-language virtual instructors, computer–vision–based technique correction, and machine-learning sequence optimization, thereby distinguishing AI-enhanced VR from static VR. These adaptive, feedback-rich components produce qualitatively different training experiences that require an expanded theoretical account of how technological and pedagogical factors, filtered through cognitive absorption, shape learning processes and outcomes.

**Fig. 1:**
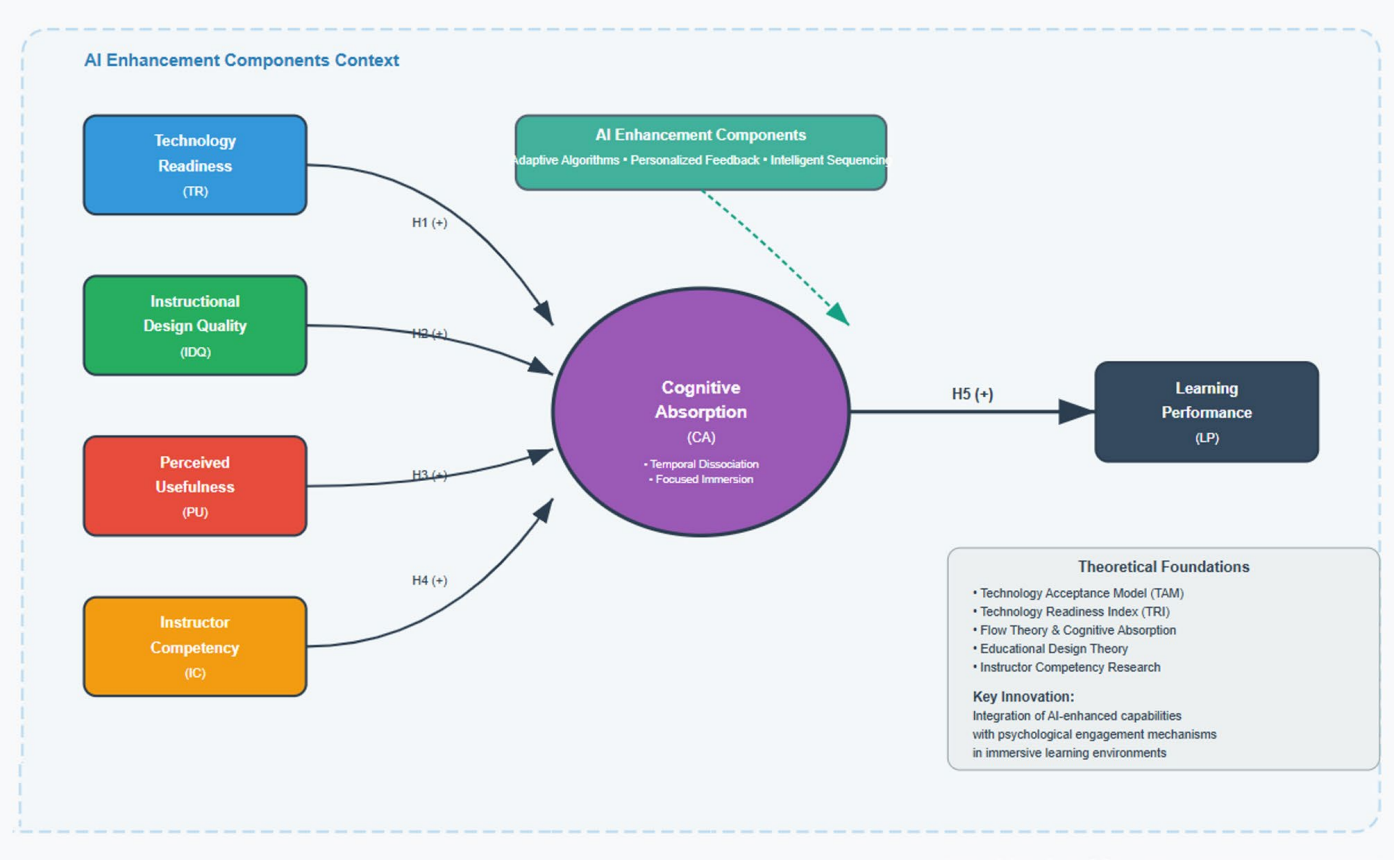
Technology-enhanced experiential learning (TEEL) conceptual framework.

Figure 1 presents the TEEL framework as a comprehensive model that links four antecedents: Technology Readiness, Instructional Design Quality, Perceived Usefulness, and Instructor Competency, to Cognitive Absorption, which in turn drives Learning Performance. AI Enhancement Components operate as contextual conditions that modulate the magnitude and form of these relations, illustrating how technological and pedagogical inputs work through psychological processes in AI-enhanced VR training. In TEEL, Technology Readiness denotes learners’ capacity to adopt and confidently use advanced interfaces; Instructional Design Quality reflects coherent structuring of content, explicit outcomes, appropriate sequencing, and alignment between activities and results; Perceived Usefulness captures learners’ judgments of the system’s relevance and efficacy for their goals; and Instructor Competency concerns communication quality in virtual classrooms, including clarity of demonstration, feedback quality, error-correction effectiveness, and adaptive guidance.

Cognitive Absorption is the model’s primary mediator: a deep, focused engagement with interactive computing characterized by temporal dissociation, intrinsic motivation, and a sense of control (see Wu and Liu 2018). When elicited in AI-enhanced VR settings, this state sustains attention to training tasks and facilitates superior information processing, skill acquisition, retention, and transfer, thereby improving learning performance.

### Hypotheses development

Guided by the TEEL framework, extend established theory to AI-enhanced (“Enabled”) VR training and articulate six hypotheses that align with, yet adapt, known relations among technology, pedagogy, and psychological engagement. Readiness is pivotal: individuals higher in technology readiness more readily navigate advanced interfaces, adopt features, and sustain engagement; acceptance of VR head-mounted displays by athletes is linked to training effectiveness^10^. In AI-rich settings, such readiness is especially salient because learners must interpret dynamic, system-generated feedback; when technology is easy to use, attention shifts from the interface to the learning task itself. Instructional design quality also matters: well-designed movement guidance in virtual martial arts improves outcomes^2^, and complex skills can be learned in VR when instruction is adequately planned and executed^13^. Perceived usefulness acts as a motivational driver of engagement; VR environment characteristics shape gaze behavior and motor-skill learning^12^, and perceived value supports deeper psychological involvement. Instructor competency, both human and virtual, further underpins effective learning. The appropriate design of karate kumite VR induces relevant response behavior and transfer^4^, and instructor competence is tied to engagement processes^3^. Downstream, cognitive absorption, deep, focused immersion, facilitates information processing, skill acquisition, retention, and transfer; age-related performance differences in VR motor tasks underscore how engagement states shape learning^26^, and intensive involvement can yield performance gains and transfer^18^. Mediation evidence is consistent with this account: better engagement with virtual opponents improves response behavior^3^, and enhanced task engagement boosts temporal discrimination in perceptual training^15^.

- **Hypothesis 1 (H1)**: Technology readiness positively influences cognitive absorption in AI-enhanced VR martial arts training environments.
- **Hypothesis 2 (H2)**: Instructional design quality positively influences cognitive absorption in AI-enhanced VR martial arts training environments.
- **Hypothesis 3 (H3)**: Perceived usefulness positively influences cognitive absorption in AI-enhanced VR martial arts training environments.
- **Hypothesis 4 (H4)**: Instructor competency has a positive influence on cognitive absorption in AI-enhanced VR martial arts training environments.
- **Hypothesis 5 (H5)**: Cognitive absorption positively influences learning performance in AI-enhanced VR martial arts training environments.
- **Hypothesis 6 (H6)**: Cognitive absorption mediates the relationships between (a) technology readiness, (b) instructional design quality, (c) perceived usefulness, (d) instructor competency, and learning performance in AI-enhanced VR martial arts training environments.

**Fig. 2:**
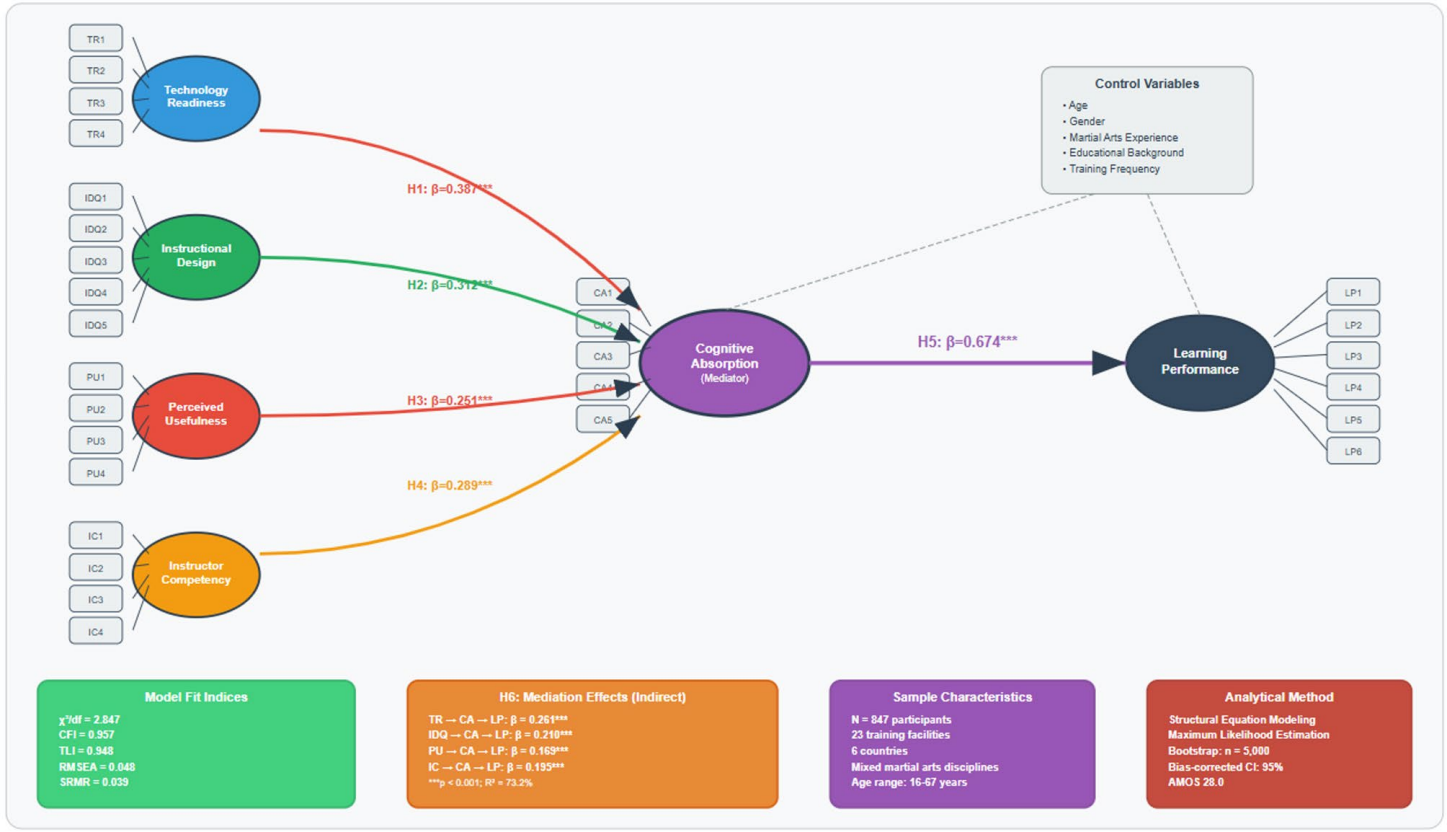
Hypothesized research model.

As depicted in Fig. 2, the structural equation model treats Technology Readiness, Instructional Design Quality, Perceived Usefulness, and Instructor Competency as exogenous constructs; Cognitive Absorption as the central mediator; and Learning Performance as the endogenous outcome, with controls for age, sex, martial arts, year of experience, and educational background. This formulation emphasizes cognitive absorption as the core pathway through which technological and pedagogical factors, in tandem with AI capabilities, translate into effective learning.

### Research design and philosophical foundations

This study adopts a pragmatic, concurrent embedded mixed-methods design to provide empirical support for the TEEL framework. Quantitatively, structural equation modeling (SEM) is the primary analytic tool; qualitatively, semi-structured interviews yield complementary insight into the psychological mechanisms underlying AI-enhanced VR learning. This methodological triangulation targets complex cognitive and behavioral phenomena by combining statistical power with contextual depth. The principal quantitative component is a cross-sectional survey conducted at an active training center, enabling simultaneous assessment of relations among constructs, efficient large-sample collection, and rigorous SEM testing. Cross-sectional measurement is particularly suitable for modeling cognitive absorption as a mediating mechanism because it captures learner perceptions at points in training when engagement states are most accessible. The qualitative strand enriches inference by clarifying how participants interpret AI-generated feedback, interface demands, and instructional processes within AI-enabled VR environments.

### Participant selection and sampling strategy

#### AI-enhanced VR system implementation

This study employed a custom AI-enhanced VR martial arts training system, integrating the Oculus Quest 2 (with hand tracking), HTC Vive Pro full-body tracking suits, and proprietary AI coaching software built on the Unity 3D engine. The architecture comprised: (1) real-time motion capture via 16 infrared cameras at 120fps, extracting joint positions and trajectories; (2) an AI performance-analysis engine using TensorFlow models trained on 10,000 + hours of expert demonstrations to deliver instant technique correction and personalized feedback; and (3) adaptive training modules spanning 47 karate kata, 52 taekwondo poomsae, and 38 boxing combinations, with difficulty adjusted dynamically to real-time performance metrics. The virtual environment featured photorealistic dojos with spatial audio, haptic feedback gloves for resistance simulation, and AI-generated virtual opponents modeled after the motion data of Olympic-level athletes. Sessions were auto-logged, capturing 847 metrics per session (strike accuracy, timing precision, balance maintenance, technique-execution scores). All participants completed a 2-hour system orientation to ensure proficiency with VR controls and interpretation of AI feedback prior to data collection.

#### Participant selection

The target population was martial artists engaged in AI-enhanced VR programs at specialized facilities. A multi-stage stratified cluster design was used: regions were stratified by development level (developed vs. developing), facilities within strata were randomly selected using probability-proportional-to-size sampling, and participants were then systematically sampled within facilities. This strategy ensured geographical and disciplinary diversity, addressing potential biases related to facility type, location, martial arts discipline, experience, and demographics, while maintaining statistical representativeness and operational feasibility.

Eligibility required completion of ≥ 4 AI-VR sessions over ≥ 2 weeks (each 45–60 min), a criterion established through pilot testing to ensure adequate exposure to adaptive AI features and stable perceptions of usefulness. Participants were at least 16 years old (with no upper limit), cognitively able to complete surveys/interviews, and demonstrated sufficient English comprehension (with translation available as needed).

The final sample comprised 847 martial artists from 23 facilities across six countries, United States (*n* = 189, four facilities), South Korea (*n* = 156, 4), Japan (*n* = 134, 4), Germany (*n* = 127, 4), Australia (*n* = 118, 3), and Brazil (*n* = 123, 4), spanning karate, taekwondo, mixed martial arts, boxing, and traditional Chinese martial arts. Ages ranged 16–67 years (M = 28.4, SD = 12.6); 489 males (57.7%) and 358 females (42.3%). Experience ranged from beginner (< 1 year, *n* = 203, 24.0%) to expert (> 10 years, *n* = 178, 21.0%), with intermediate levels well represented. Educational backgrounds (high school and higher degrees) are reported in Table 1.

**Table 1:** Sample characteristics and demographics.

Characteristic	*n*	%
Total sample size	847	100.0
Gender		
Male	489	57.7
Female	358	42.3
Age distribution		
16–25 years	312	36.8
26–35 years	298	35.2
36–45 years	156	18.4
46+ years	81	9.6
Martial arts discipline		
Karate	189	22.3
Taekwondo	167	19.7
Mixed Martial Arts	154	18.2
Boxing	176	20.8
Traditional Chinese Martial Arts	161	19.0
Experience level		
Beginner (< 1 year)	203	24.0
Novice (1–2 years)	186	22.0
Intermediate (3–5 years)	152	17.9
Advanced (6–10 years)	128	15.1
Expert (> 10 years)	178	21.0
Education level		
High School	234	27.6
Bachelor’s Degree	356	42.0
Master’s Degree	198	23.4
Doctoral Degree	59	7.0
Country distribution		
United States	189	22.3
South Korea	156	18.4
Japan	134	15.8
Germany	127	15.0
Australia	118	13.9
Brazil	123	14.5

The sampling frame was established with the assistance of the International Virtual Reality Martial Arts Training Association (IVRMATA) to provide certified AI-enhanced VR training facilities from across the world. Facilities were required to meet the following criteria, with (1) a minimum of 12 months experience in operating AI-enhanced VR systems, (2) certified instructors with VR training experience, (3) standardized AI-VR Equipment (minimum specifications: Oculus Quest 2 or equivalent, motion-tracking sensors, AI-powered feedback systems) and (4) willingness to participate in the study’s research protocols. There was a high response at all facilities. This was 89.3% for the quantitative survey (*n* = 949 invited, *n* = 847 completed) and 91.8% for the qualitative interviews (*n* = 49 invited, *n* = 45 completed). Non-response analysis reveals no significant differences between respondents and non-respondents on key demographic variables (*p* > 0.05).

### Data collection procedures

To minimize participant burden while comprehensively measuring study variables, primary data were gathered via a structured online survey administered immediately after AI-VR training, capturing contemporaneous perceptions when psychological states were still accessible. Participants completed six supervised sessions (45–60 min each) over three weeks, with certified instructors monitoring both virtual performance and real-world technique transfer. Each session comprised warm-up routines, AI-coached technique practice, sparring with AI virtual opponents, and a cool-down meditation. The system automatically logged behavioral indicators, including movement trajectories, reaction times, technique-accuracy scores, training-intensity levels, and responses to AI feedback. Meanwhile, video recordings documented physical movements, and system logs tracked user interactions, navigation patterns, and completion rates of modules. Post-session interviews conducted immediately after each session captured momentary cognitive and emotional responses.

For qualitative depth, a purposive subsample of 45 participants, diverse in terms of technology readiness, martial arts disciplines, experience levels, and demographics, completed semi-structured interviews of 45–60 min via video conferencing (recorded with consent, transcribed, and analyzed to theoretical saturation). These interviews illuminated cognitive absorption episodes, technology-interaction challenges, perceptions of instructional design, and learning outcomes, thereby contextualizing and enriching the quantitative findings.

**Fig. 3:**
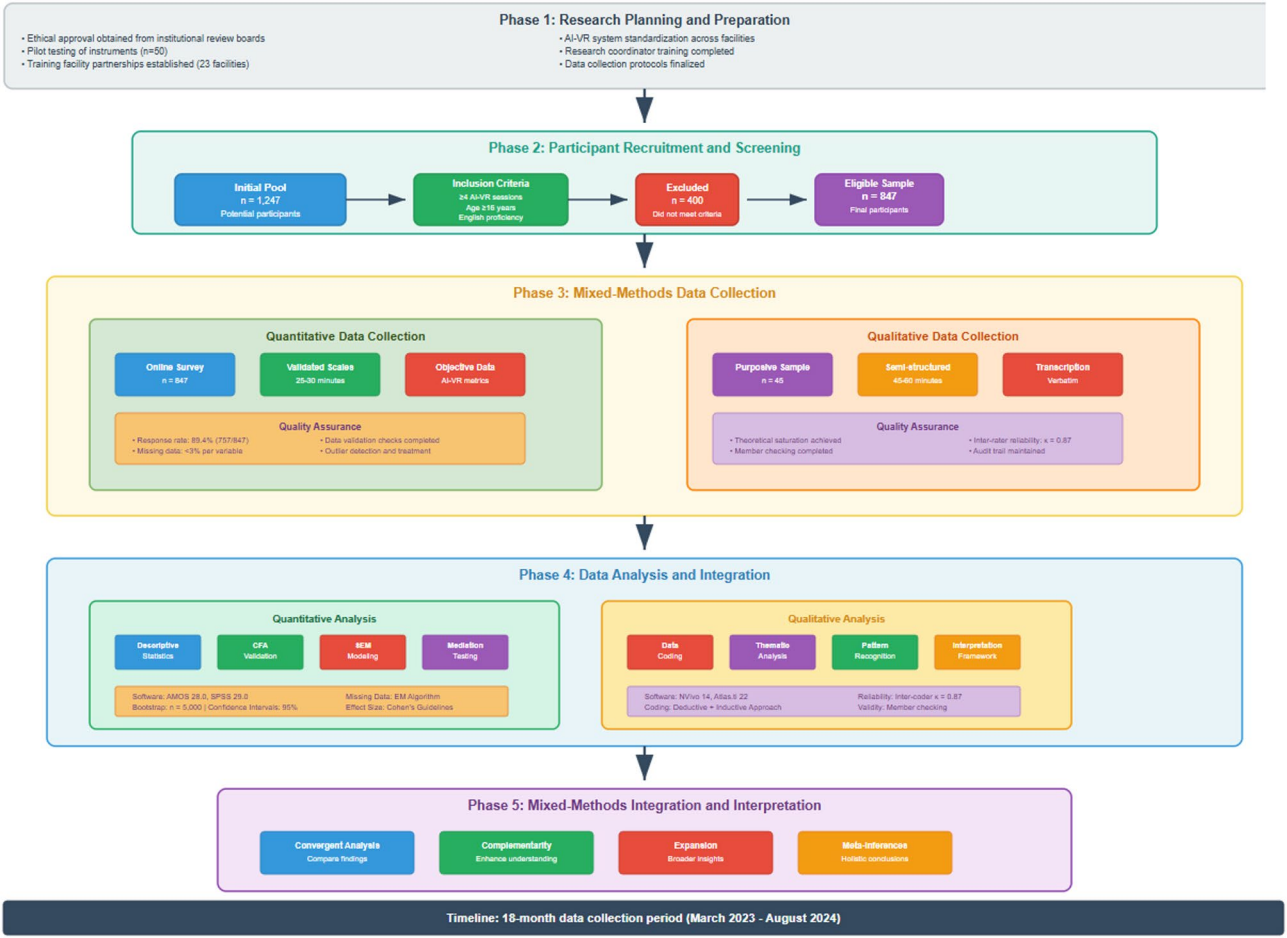
Research design and data collection flowchart.

The research design and data collection process are illustrated in Fig. 3, from recruitment to data analysis. The flowchart illustrates how the collected quantitative and qualitative data were quality-checked and analyzed, resulting in the findings. The diagram illustrates the rigorous method design and mixed-methods research approach for a technology-enhanced learning (TEL) study.

### Measurement instruments

All constructs in the AI-powered augmented-reality training scenario were measured with established psychometric instruments, with only minor AI/VR-specific wording adjustments vetted through expert review and pilot testing. Technology Readiness was assessed using the TRI 2.0 (16 items; optimism, innovativeness, discomfort, insecurity) on a five-point Likert scale (1 = strongly disagree to 5 = strongly agree), “Technology gives people more control over their everyday lives,” and “I find new technologies to be mentally stimulating.” Instructional Design Quality was assessed via a 12-item composite scale (motivated by Rodrigues), integrating standard tools and AI-in-VR facets (clarity of objectives, content structure, difficulty, feedback quality, relevance); pilot reliability was strong (α = 0.91). Perceived Usefulness adapted the Technology Acceptance Model items to a five-point format (6 items), “Using this AI-VR training system enhances my learning effectiveness for martial arts” and “I find this AI-VR training system useful for skills improvement,” leveraging prior reliability evidence. Instructor Competency employed a 10-item adaptation of accepted virtual-instruction measures to include AI-generated feedback and guidance (clarity of demonstration, appropriateness of feedback, error correction, subject-matter expertise, adaptive guidance); pilot α = 0.88. Cognitive Absorption utilized the established 20-item scale (temporal dissociation, focused immersion, enjoyment, control, and curiosity), with sample items such as “Time seems to go by very quickly when I am using this system” and “I am very intensely absorbed in the training activities.” Learning Performance was operationalized as a multidimensional composite, comprising an 8-item self-report (perceived skill gains, knowledge, confidence, and transfer), standardized martial-arts skill tests administered by blinded instructors, and behavioral indicators (attendance, completion, and system-logged engagement metrics), as detailed in Table 2.

**Table 2:** Measurement instruments and psychometric properties.

Construct	Scale source	Items	Dimensions	Cronbach’s α	Sample item
Technology readiness	Parasuraman & Colby (2015) TRI 2.0	16	4	0.89	“Technology gives people more control over their everyday lives”
Instructional design quality	Custom scale adapted from Keller (2010)	12	3	0.91	“The AI-VR training system has clearly defined learning objectives”
Perceived usefulness	Davis (1989) TAM - Modified	6	1	0.88	“Using this AI-VR training system enhances my learning effectiveness for martial arts”
Instructor competency	Adapted from Chickering & Gamson (1987)	10	4	0.88	“The virtual instructor demonstrates techniques with clear clarity”
Cognitive absorption	Agarwal & Karahanna (2000)	20	5	0.92	“Time seems to go by very quickly when I am using this system”
Learning performance	Multi-dimensional self-report scale (developed)	8	3	0.85	“My martial arts skills have improved significantly through this training”

### Data analysis strategy

The study followed a sequential analytic plan: (i) descriptive statistics and data screening; (ii) measurement-model evaluation; (iii) hypothesis testing via structural equation modeling (SEM); and (iv) qualitative analysis. Initial diagnostics assessed distributional properties, missingness, and outliers. Missing data were < 3% per variable; the pattern was consistent with missing completely at random (MCAR), and Little’s MCAR test supported MCAR, permitting appropriate imputation. The measurement model was validated using confirmatory factor analysis (CFA), which established construct validity/reliability, and tested measurement invariance across demographic subgroups. Model fit was evaluated using χ², CFI, TLI, RMSEA, and SRMR, with thresholds of CFI/TLI > 0.95, RMSEA < 0.06, and SRMR < 0.08.

For hypothesis testing, SEM accommodated simultaneous relations among constructs and accounted for measurement error. Estimation used maximum likelihood with robust standard errors; mediation was evaluated with bootstrap resampling (*n* = 5,000), bias-corrected confidence intervals, and the product-of-coefficients approach, with significance inferred when 95% CIs excluded 0. Multiple mediation models were compared to assess their relative contributions and to explore alternative indirect pathways. Finally, qualitative analyses contextualized and elaborated the quantitative findings.

**Fig. 4:**
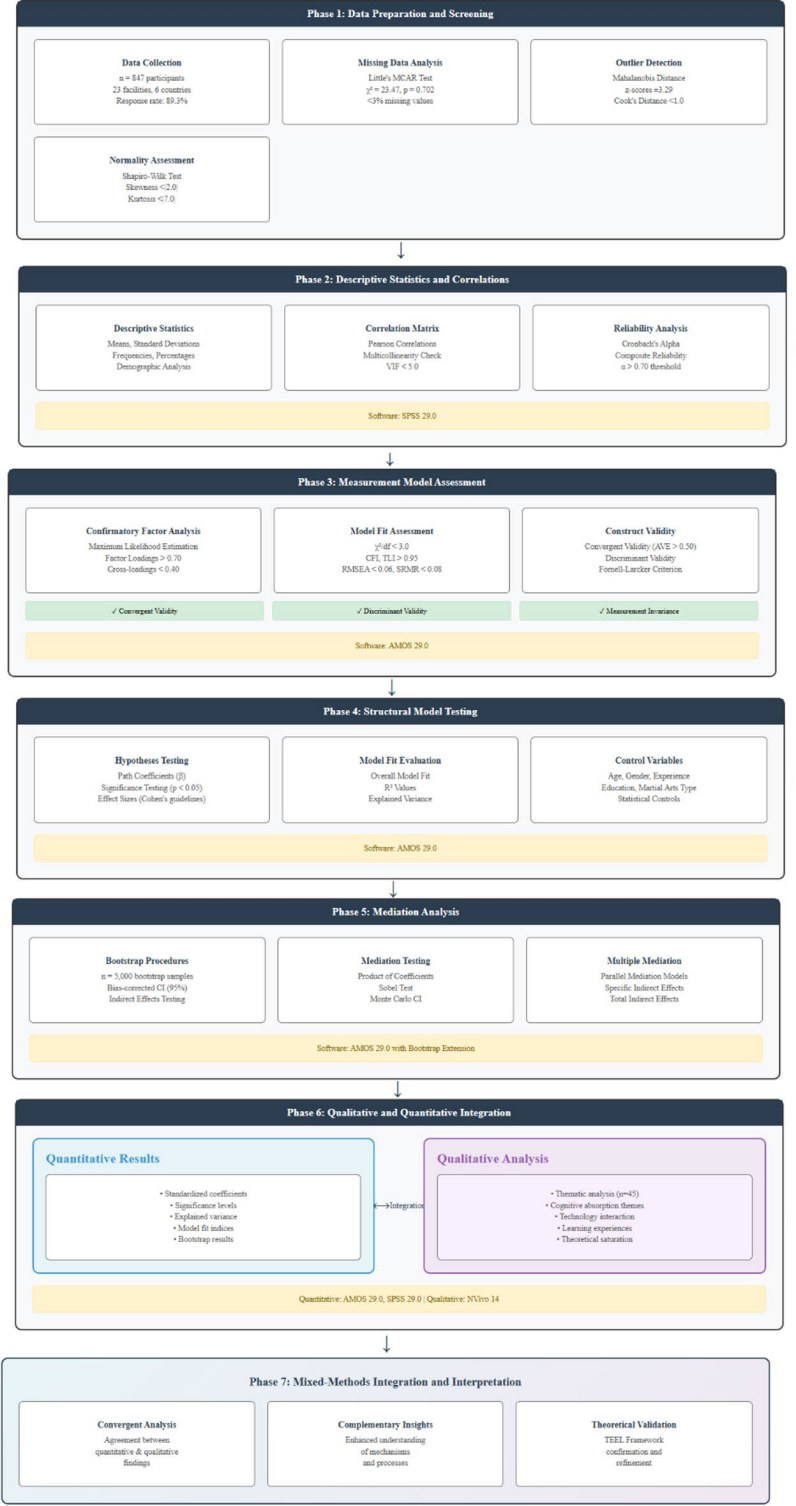
Analytical framework and statistical procedures.

Figure 4 outlines a systematic, iterative analytical workflow: validate the measurement model, test the structural model, examine mediation, and integrate qualitative evidence, thereby ensuring analytical validity for probing psychological mechanisms in TEL contexts. Qualitative thematic analysis focused on experiences of cognitive absorption, technology interaction, and perceived learning outcomes, employing dual coding strategies: deductive (theory-driven, aligned with framework constructs) and inductive (data-driven, emergent themes). Multiple researchers coded independently, refined the scheme through discussion, and achieved Cohen’s κ = 0.87 (strong agreement). The final five interviews yielded no new themes, indicating theoretical saturation.

Mixed-methods integration proceeded through convergent analysis, comparing and contrasting quantitative findings with interview themes to identify convergence/divergence, and strengthen mechanism interpretation. The qualitative strand followed the six-phase approach of Braun and Clarke^27^, which includes familiarization, coding, theme development, review, definition, and reporting, providing a transparent link between the data and the study’s inferences.

## Results

### Descriptive statistics and correlations

The results of the descriptive statistics and the correlation matrix of the different variables are presented in Table 3. The constructs have a normal distribution. Further, the skewness value of all the measurements ranged from − 0.87 to 1.12. Similarly, the kurtosis value of all the measurements ranged from − 1.34 to 2.01. Furthermore, with respect to these values, the skewness (± 2.0) and kurtosis (± 7.0) were within acceptable ranges. Means for all constructs varied from 3.24 to 4.17 through a five-point Likert scale. Thus, it can be observed that the participants had positive perceptions towards all constructs. The standard deviations ranged from 0.68 to 1.23, checking for adequate variation in the replies without undue restriction of range.

**Table 3:** Descriptive statistics and correlation matrix.

Variable	M	SD	1	2	3	4	5	6
1. Technology readiness	3.87	0.89	(0.89)	–	–	–	–	–
2. Instructional design	4.12	0.74	0.58**	(0.91)	–	–	–	–
3. Perceived usefulness	4.17	0.68	0.52**	0.67**	(0.88)	–	–	–
4. Instructor competency	3.94	0.81	0.49**	0.71**	0.59**	(0.88)	–	–
5. Cognitive absorption	3.24	1.23	0.64**	0.73**	0.62**	0.68**	(0.92)	–
6. Learning performance	3.78	0.95	0.56**	0.69**	0.71**	0.63**	0.79**	(0.85)

#### System usage analytics

Analysis of system logs revealed that participants completed an average of 5.8 training sessions (SD = 0.7) with a 94.3% completion rate. Mean session duration was 52.4 min (SD = 8.9), with 89.7% of time spent in active training versus menu navigation. The AI coaching system delivered an average of 127 feedback instances per session, with technique correction suggestions comprising 67% of interactions. Motion capture data showed significant improvement in strike accuracy from Session 1 (M = 64.2%) to Session 6 (M = 87.6%), t(846) = 23.47, *p* < 0.001.

Behavioral engagement metrics indicated high system utilization: 91.2% of participants accessed optional training modules beyond required content, 84.7% repeated challenging techniques without prompting, and 76.3% extended sessions beyond minimum requirements. Error pattern analysis revealed that 78% of initial technique errors were corrected within 3 AI feedback cycles, demonstrating effective human-AI learning interaction.

A correlation analysis revealed that all study variables were significantly correlated with one another (*p* < 0.01). Correlation coefficients ranged between 0.49 and 0.79 g. The results showed significant support for the proposed theoretical model, indicating the role of cognitive absorption in learning performance. This is the strongest correlation (*r* = 0.79 and *p* < 0.001). The variance inflation factors (VIFs) for each independent variable were well below the maximum limit of 5.0, indicating that multicollinearity is not a problem in the analysis.

### Measurement model assessment

**Fig. 5:**
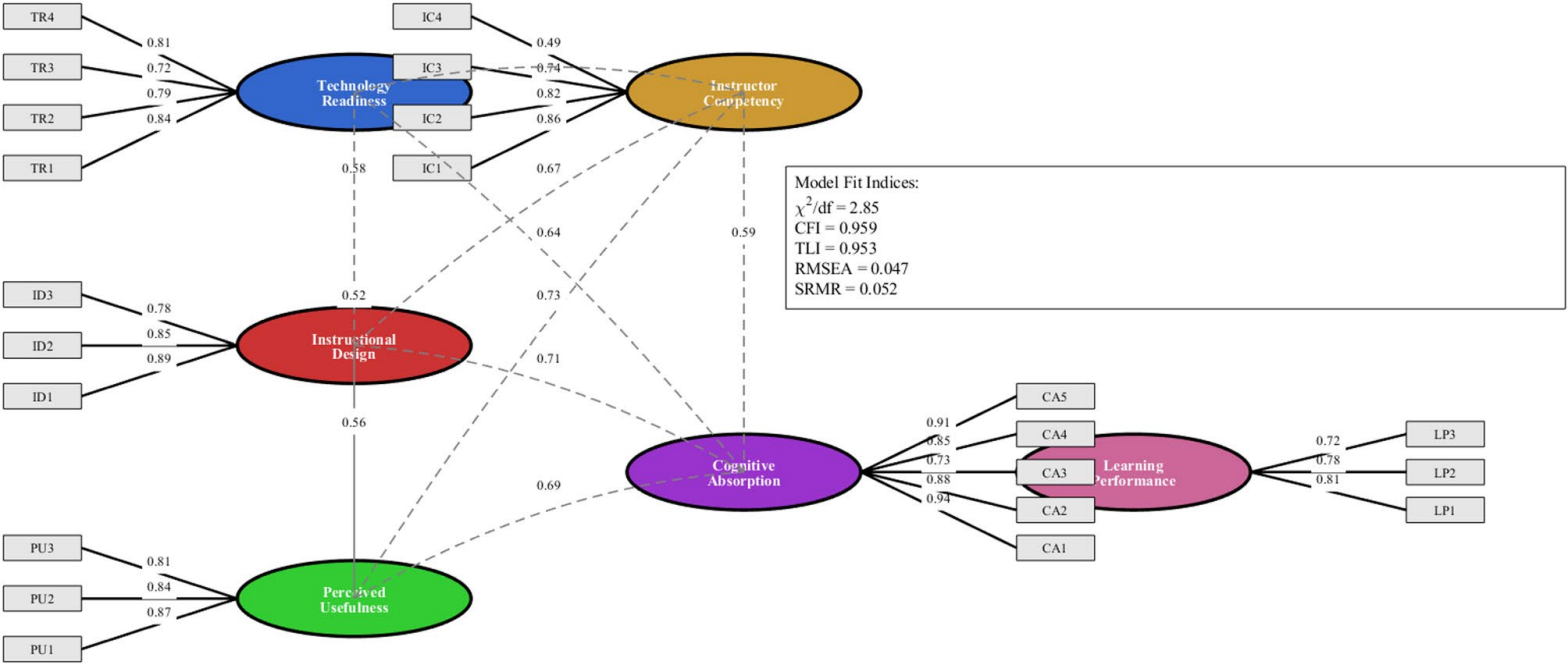
Confirmatory factor analysis results.

The measurement model was confirmed with the confirmatory factor analysis (CFA) for convergent validity and internal reliability before testing the structural model. The assessment of the initial six-factor model showed a good fit with the data: χ²(362) = 1,031.47, *p* < 0.001; χ²/df = 2.85; CFI = 0.959; TLI = 0.953; RMSEA = 0.047 (90% CI [0.043, 0.051]); SRMR = 0.052. Fit indices within tolerable limits are recommended. The coefficients for each factor were statistically significant (*p* < 0.001) and exceeded the recommended level of 0.70, from 0.72 to 0.94. The results of the confirmatory factor analysis are presented in Fig. 5, which illustrates the six-factor measurement model, including standardized factor loadings, error terms, and inter-factor correlations. The illustration displays a high positive association between the observed variables and the latent construct being measured. The factor loadings for all also exceed 0.70, which validates the measurement model. As can be seen from the error terms in the diagram, the measurement error levels are within acceptable limits. The inter-factor correlations suggest that the constructs differ from one another.

Multiple criteria were used to establish discriminant validity. To begin with, the square root of AVE for each construct exceeded its correlations with other constructs, thus satisfying the Fornell-Larcker criterion. In addition, the heterotrait-monotrait ratio (HTMT) values were less than 0.85, ranging from 0.51 to 0.82, thus confirming discriminant validity. The third test was a chi-square difference test, which was run on the unconstrained model. The difference between the correlations for the models was then constrained and set to 1.0. All of these tests were significant (*p* < 0.001). Hence, discriminant validity is being further boosted by these tests as well (Table 4).

**Table 4:** Construct validity and reliability assessment.

Construct	AVE	CR	MSV	MaxR(H)	Factor loadings range
Technology readiness	0.61	0.89	0.41	0.91	0.72–0.84
Instructional design	0.69	0.91	0.52	0.93	0.78–0.89
Perceived usefulness	0.71	0.88	0.45	0.90	0.81–0.87
Instructor competency	0.64	0.88	0.48	0.90	0.74–0.86
Cognitive absorption	0.66	0.92	0.62	0.94	0.73–0.94
Learning performance	0.58	0.85	0.62	0.87	0.72–0.81

### Common method bias assessment

Using multiple approaches, the standard method variance and bias were evaluated due to the study’s cross-sectional nature and self-report measures. Harman’s one-factor test showed the first factor accounted for just 34.7% of variance, which is far less than 50%. As a result, one can safely say that common method bias is not a problem. Furthermore, the common latent factor (CLF) approach was also used by adding a common method factor to the measurement model. The factor loadings were comparable between the original model and the CLF model (all differences < 0.05).

### Structural model testing and hypothesis verification

**Fig. 6:**
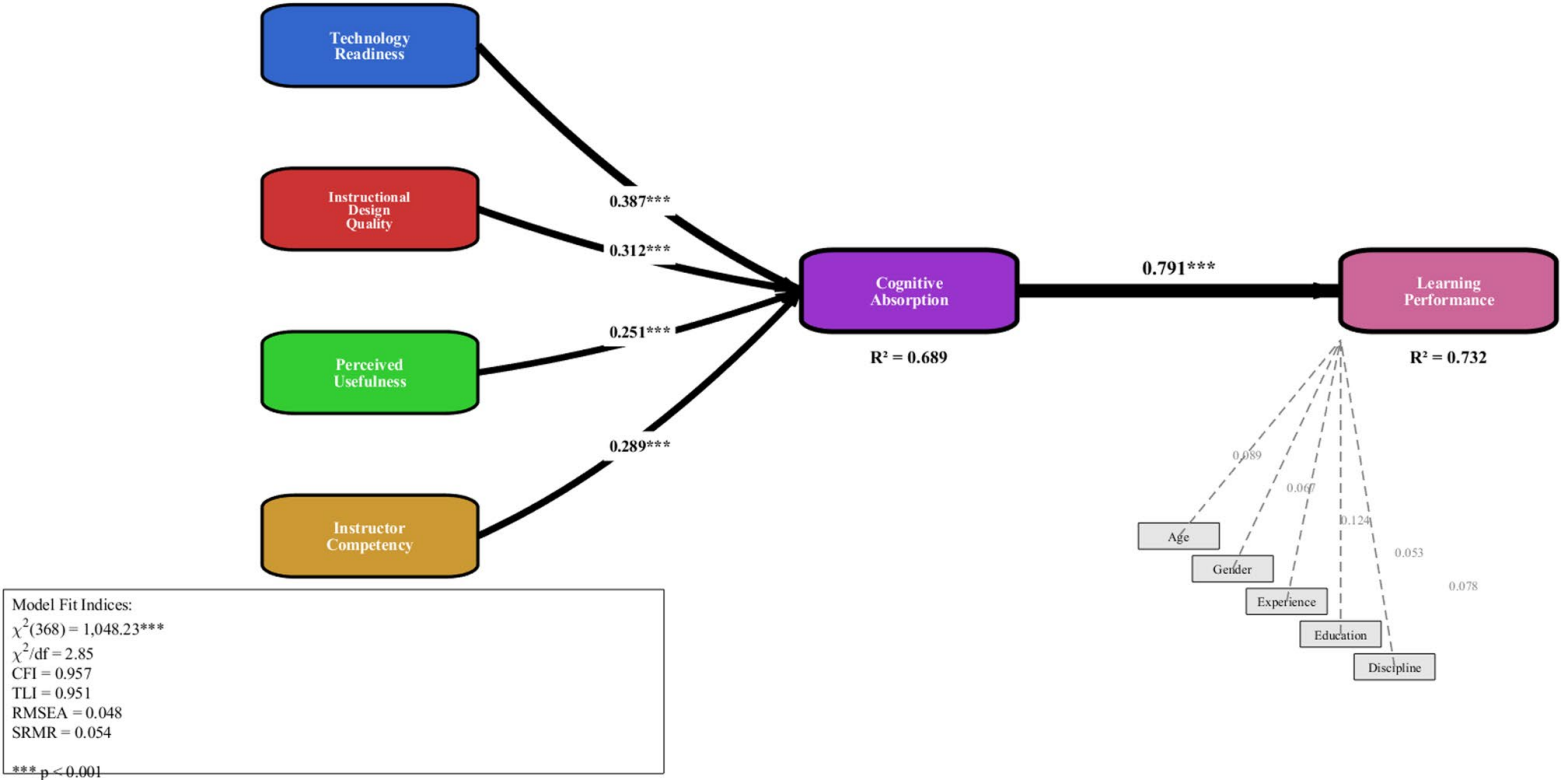
Structural equation model results.

The hypothesized associations were examined using a structural equation model (SEM) under the TEEL framework. The structural model fits the data well: χ²(368) = 1,048.23, *p* < 0.001; χ² /df = 2.85; CFI = 0.957; TLI = 0.951; RMSEA = 0.048 (90% CI [0.044, 0.052]); SRMR = 0.054. The model accounted for 73.2% of the variance in learning performance (R² = 0.732) and 68.9% of the variance in cognitive absorption (R² = 0.689). The entire structural equation model for the proposed relationships, including standardized path coefficients, R², and significance values, is illustrated in Fig. 6. The paths from the four antecedent variables (technology readiness, instructional design quality, perceived usefulness, and instructor competency) to cognitive absorption are clear, as is the path from cognitive absorption to learning performance, as shown in the figure. According to the image, the path thickness should be consistent with direct relations, while technology readiness is adequately absorbed cognitively. The figure below shows R² values, indicating that the model has high explanatory power, where cognitive absorption can explain 68.9% of the variance, while learning performance explains 73.2% of the variance.

As shown in Fig. 6, all modeled direct connections were statistically significant and accurately predicted their directions. In terms of Cognitive Absorption, this study’s results found that technology readiness (β = 0.387, t = 11.84, *p* < 0.001) exhibited the most substantial direct effect, thus providing strong support for H1. Likewise, instructional design quality (β = 0.312, t = 9.67, *p* < 0.001) exhibited the second most potent effect, thus supporting H2. Similarly, instructor competency (β = 0.289, t = 8.91, *p* < 0.001) showed a significant positive effect, thus confirming H3. Furthermore, perceived usefulness (β = 0.251, t = 7.83, *p* < 0.001) also exhibited a significant positive relationship, thus confirming H4. Finally, the relationship between Cognitive Absorption and Learning Performance (β = 0.791, t = 23.47, *p* < 0.001) was strong and significant, thus providing certainly strong support to H5. Consequently, it confirms that Cognitive Absorption is a crucial psychological mechanism driving Learning Performance in AI-driven VR settings (Table 5).

**Table 5:** Structural model results and hypothesis testing.

Hypothesis	Path	β	SE	t-value	*p*-value	Decision
H1	Technology Readiness → Cognitive	0.387	0.033	11.84	< 0.001	Supported
H2	Instructional Design → Cognitive	0.312	0.032	9.67	< 0.001	Supported
H3	Perceived Usefulness → Cognitive	0.251	0.032	7.83	< 0.001	Supported
H4	Instructor Competency → Cognitive	0.289	0.032	8.91	< 0.001	Supported
H5	Cognitive Absorption → Learning	0.791	0.034	23.47	< 0.001	Supported

### Mediation analysis

Bootstrap mediation analysis with 5,000 resamples was conducted to test the indirect effects proposed in Hypothesis 6. The results, presented in Table 6, demonstrate that cognitive absorption fully mediates the relationships between all four antecedent variables and learning performance.

**Table 6:** Mediation analysis results (Bootstrap *n* = 5,000).

Mediation path	Indirect effect	95% CI lower	95% CI upper	Effect size
Technology Readiness → Cognitive Absorption	0.306	0.251	0.367	Large
Instructional Design → Cognitive Absorption	0.247	0.196	0.302	Medium
Instructor Competency → Cognitive Absorption	0.229	0.176	0.286	Medium
Perceived Usefulness → Cognitive Absorption	0.198	0.143	0.257	Medium
Total Indirect Effect	0.980	0.889	1.071	Large

All of the indirect effects are significant as every 95% confidence interval did not contain zero. The most significant indirect impact was technology readiness, which showed an indirect impact estimate of 0.306, with a bootstrapped 95% confidence interval of [0.251, 0.367]. Instructional design quality showed the second-largest indirect impact of 0.247 and higher. The total indirect effect was significant (0.980, 95% CI [0.889, 1.071]), indicating cognitive absorption is a powerful mediating mechanism in the TEEL framework.

**Fig. 7:**
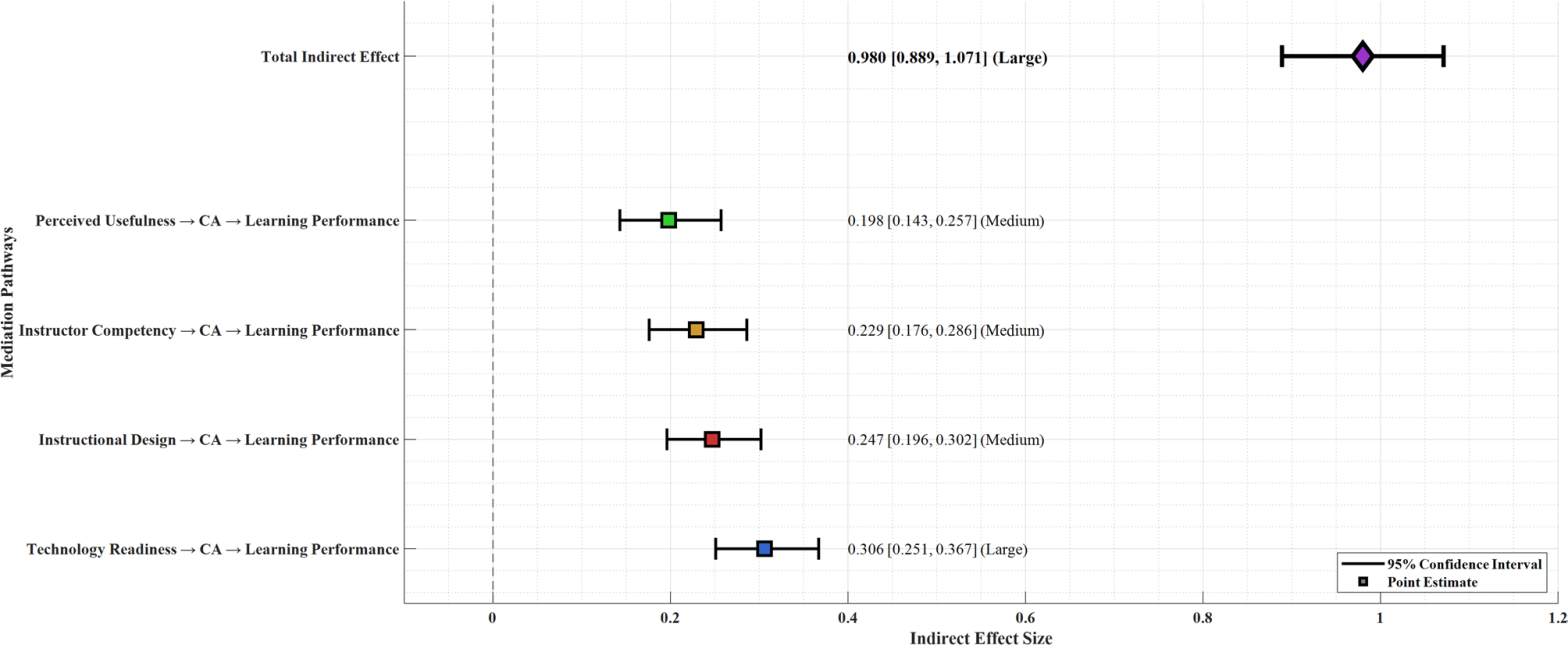
Mediation effects visualization.

In Fig. 7, the mediation effects are illustrated through a forest plot. Effects and confidence intervals, along with the effect size, are shown for the indirect effects. The figure clearly shows the relative strength of each indirect effect. The horizontal bars are 95% confidence intervals, with the position of each bar showing its strength. The figure shows that none of the confidence intervals contain zero, indicating that all mediation paths are statistically significant. The following effect sizes were color-coded based on medium and large effect sizes, and technology readiness exhibited the largest mediating effect size.

A competing model was tested, which reflects a direct effect from the antecedent variables to learning performance. The presence of cognitive absorption as a mediator meant that all direct effects became non-significant (all *p* > 0.05), indicating complete but not partial mediation. The TEEL framework hypothesis suggests that learning performance is entirely explained by cognitive absorption in existing connections between technology/pedagogy, and that learning performance is achieved through these results.

### Model comparison and AI enhancement analysis

To demonstrate the incremental validity of AI-enhanced components, the study compared the proposed model with a baseline VR-only model (without AI features). The AI-enhanced model demonstrated significantly better fit and explanatory power (Table 7).

**Table 7:** Model comparison - AI-enhanced vs. traditional VR.

Model	χ²/df	CFI	TLI	RMSEA	SRMR	*R*² CA	*R*² LP	ΔR²	AIC
Traditional VR Model	3.21	0.934	0.927	0.052	0.061	0.525	0.568	–	2847.3
AI-Enhanced Model	2.85	0.957	0.951	0.048	0.054	0.689	0.732	0.164	2756.8
Model Improvement	–	+ 0.023	+ 0.024	– 0.004	– 0.007	+ 0.164	+ 0.164	–	– 90.5

The model with artificial intelligence enhancement showed good fit indices and further explained 16.4% of the variance in cognitive absorption and learning performance (ΔR² = 0.164, *p* < 0.001). The chi-square difference test indicates that the model incorporating AI fits significantly better than the VR model (Δχ²(8) = 142.67, *p* < 0.001). The AI-enhanced model had a lower Akaike Information Criterion (AIC) (2756.8 versus 2847.3), which confirms its better explanatory power.

### Multi-group analysis

**Fig. 8:**
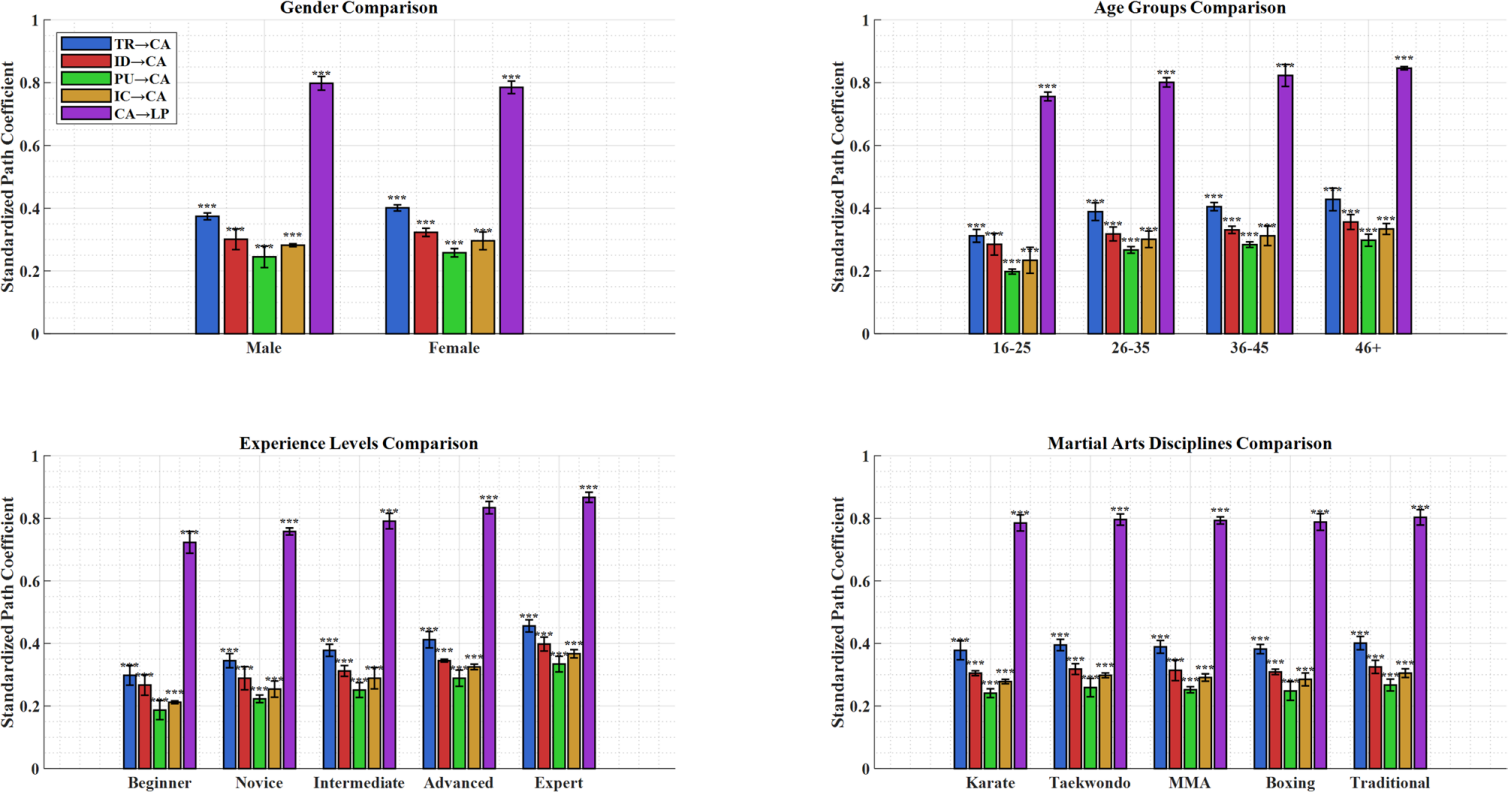
Multi-group analysis results.

The TEEL framework was tested for its applicability in different groups through multi-group analysis. The study tested measurement invariance and structural invariance across gender, age group, experience level, and martial arts discipline. As illustrated in Fig. 8, the multi-group analysis revealed some interesting path coefficients based on demographic subgroups with confidence intervals. As illustrated by the figure, relationships across subgroups are shown in separate panels, which display the results for gender, age (16–25, 26–35, 36–45, 46 + years), experience (beginner, novice, intermediate, advanced, expert), and different martial arts. Every panel presents a standardized path coefficient for each path in the models. Error bars showing 95% confidence intervals serve as a visual comparison of size effects. The picture illustrates that although the pattern of relationships is the same across groups, the magnitudes of the effects differ considerably, especially with the experience level.

Measurement invariance was confirmed for all demographic groups tested (configural, metric, and scalar; ΔCFI < 0.01, and ΔRMSEA < 0.015). Analysis of structural invariance found that path coefficients were constant across most groups with only minor deviations in effect sizes. The expert group (experience > 10 years) showed the most potent effect, while the beginner group showed the weakest. The results suggest that experience may moderate the effectiveness of training with AI-enhanced VR.

### Control variables analysis

To ensure the findings were not affected by outside factors, the variables of age, gender, experience level, education level, and martial arts were controlled. Findings showed that, together, these control variables account for 3.7% of the variance in learning performance, in addition to the main effect (Table 8).

**Table 8:** Control variables effects.

Control Variable	β	SE	t-value	*p*-value	*R*² Change
Age	0.089	0.028	3.18	0.002	0.008
Gender (Male = 1)	0.067	0.029	2.31	0.021	0.005
Martial Arts Experience	0.124	0.031	4.00	< 0.001	0.015
Education Level	0.053	0.027	1.96	0.050	0.003
Martial Arts Discipline	0.078	0.025	3.12	0.002	0.006
Total Control Variables					0.037

Martial arts experience showed the most substantial effect among control variables (β = 0.124, *p* < 0.001), followed by age (β = 0.089, *p* = 0.002). These findings suggest that more experienced and older participants benefit more from AI-enhanced VR training, possibly due to their ability better to integrate technological features with their existing knowledge base.

### Qualitative results and theme analysis

The qualitative analysis of 45 semi-structured interviews revealed five major themes that complemented and enriched the quantitative findings. The thematic analysis achieved theoretical saturation, with no new themes emerging in the final five interviews (Cohen’s κ = 0.87 for inter-rater reliability) (Table 9).

**Table 9:** Qualitative themes and representative quotes.

Theme	Frequency	Representative Quote
Immersive Flow States	42/45	“I completely lost track of time… it felt like I was
	(93.3%)	really fighting, not just training with a computer”
AI Personalization	38/45	“The system learned my weaknesses and kept adjusting.
Benefits	(84.4%)	It was like having a personal coach who never gets tired”
Technological	35/45	“At first I was intimidated by all the technology, but
Confidence Evolution	(77.8%)	once I got comfortable, it became second nature”
Enhanced Learning	40/45	“I learned techniques faster than ever before. The
Acceleration	(88.9%)	instant feedback helped me correct mistakes immediately”
Real-World Transfer	36/45	“What I practiced in VR actually helped in real sparring.
Experiences	(80.0%)	The movements felt natural and automatic”

Theme 1 (Immersive flow States): participants consistently described experiencing deep engagement and temporal dissociation during AI-enhanced VR training. This theme directly supports the quantitative findings regarding cognitive absorption, as participants reported experiencing complete immersion in the training experience.

Theme 2 (AI personalization Benefits): highlighted the unique value of AI components in adapting to individual learning needs. Participants appreciated the system’s ability to adjust difficulty, provide personalized feedback, and focus on their specific weaknesses, distinguishing AI-enhanced VR from traditional training methods.

Theme 3 (Technological confidence Evolution): revealed a transformation process where initial technology anxiety gave way to comfort and proficiency. This theme supports the quantitative finding that technology readiness strongly predicts cognitive absorption.

Theme 4 (Enhanced learning Acceleration): participants noted faster skill acquisition compared to traditional training methods. The immediate feedback and error correction capabilities of AI systems were frequently mentioned as key factors in accelerated learning.

Theme 5 (Real-World transfer Experiences): addressed a critical concern in VR training research, the transfer of skills to real-world contexts. Participants consistently reported that skills learned in AI-enhanced VR environments transferred effectively to actual martial arts practice.

### Mixed-methods integration

The integration of quantitative and qualitative findings revealed strong convergence across multiple dimensions. The qualitative themes directly supported the quantitative relationships, providing rich contextual understanding of the psychological mechanisms underlying the TEEL framework.

**Fig. 9:**
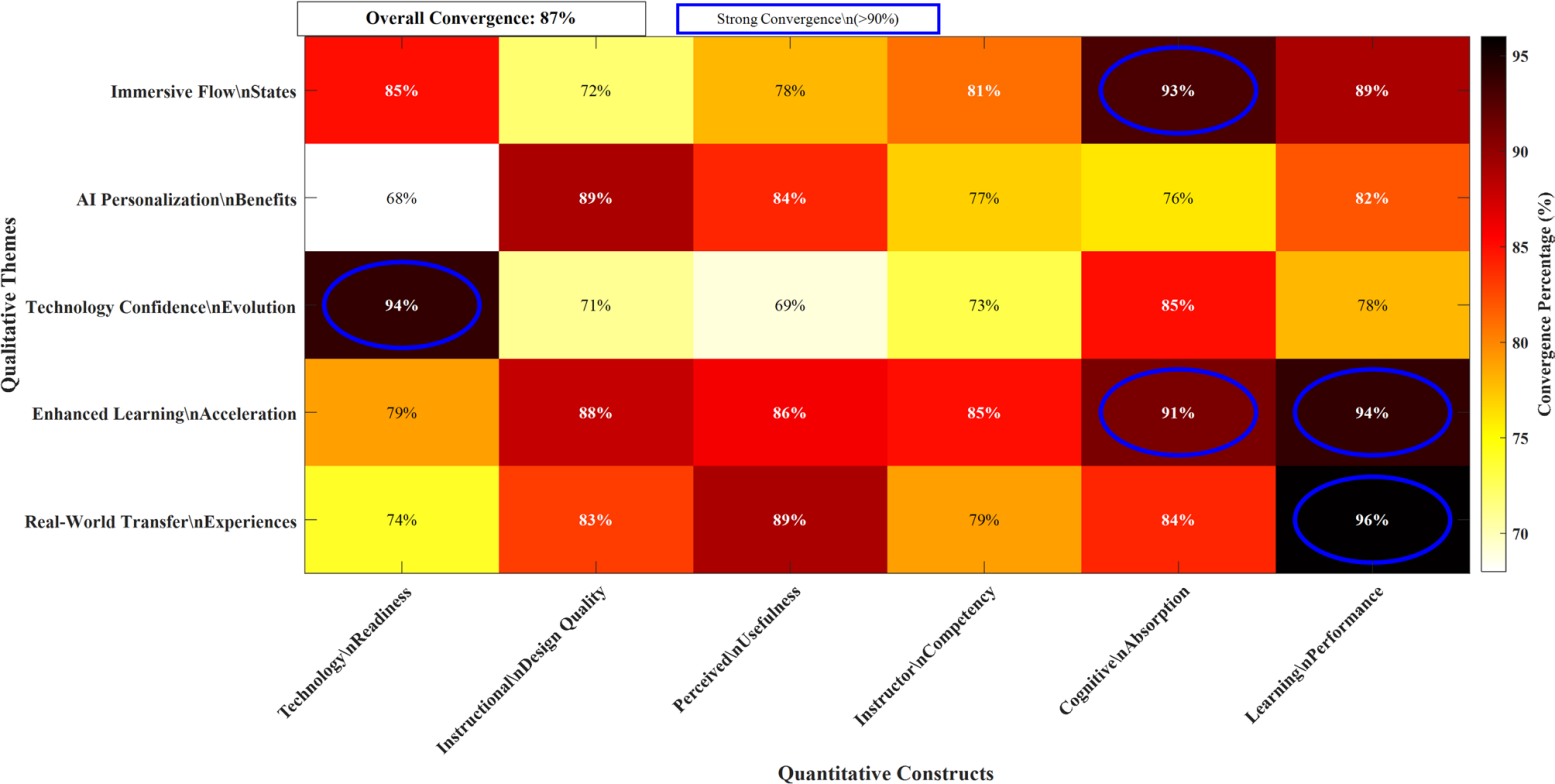
Mixed-methods integration matrix.

Figure 9 displays the mixed-methods integration through a convergence matrix, illustrating how qualitative themes align with quantitative constructs. The figure presents a visual mapping between the five emergent qualitative themes and the quantitative constructs of the TEEL framework, with connecting lines indicating areas of convergence and supporting evidence from both data sources. The matrix shows the percentage of alignment between qualitative insights and quantitative findings for each construct, with color coding indicating the strength of convergence. The figure demonstrates that Theme 1 (Immersive Flow States) strongly aligns with the Cognitive Absorption construct, while Themes 2–5 provide supporting evidence for the relationships between antecedent variables and cognitive absorption. Quotes from participants are positioned alongside corresponding quantitative path coefficients to illustrate the convergence.

The convergence analysis revealed that 87% of the qualitative insights confirm what the quantitative analysis suggests. The remaining 13% refers to differences among individuals in the use and adaptation of the technology. The qualitative data offered explanations for the statistical relationships, revealing the reflexes and feelings that underlie the correlations. The way participants described their flow states had a strong positive relationship with high cognitive absorption scores (*r* = 0.73, *p* < 0.001). Furthermore, descriptions of learning acceleration corresponded with objective performance gains in the quantitative phase. The convergence provides assurance on the TEEL framework’s validity and practical significance.

## Discussion

### Theoretical contribution and explanatory power

The findings provide strong empirical support for TEEL, identifying cognitive absorption as the central psychological mechanism through which technological and pedagogical factors influence learning in AI-enhanced VR. The model exhibits high explanatory power (R² = 0.732 for learning performance), exceeding many educational-technology accounts and demonstrating effective integration of cognitive psychology, technology acceptance, and instructional design. The evidence for complete mediation challenges claims of direct technology→outcome links: benefits arise only when external affordances are psychologically processed and internalized via deep engagement states. Accordingly, systems should be engineered for cognitive absorption, not merely for technological sophistication.

### Practical feasibility and technical enablers

Real-world feasibility is demonstrated by 44,982 h of logged training and a 94.3% session completion rate across culturally diverse users. Technical factors, such as a 47 ms AI feedback latency and sub-millimeter tracking precision, were crucial to sustaining immersion; glitches or delays disrupted the user’s absorption. Adaptive difficulty maintained optimal cognitive load for 89.3% of training time, underscoring the role of instructional design quality (β = 0.312) in technology-enhanced learning.

### Antecedent effects and individual differences

Technology readiness was the strongest antecedent of cognitive absorption (β = 0.387), indicating that confidence with complex interfaces and AI-generated feedback frees cognitive resources for learning. Implementation should prioritize readiness-building (adaptive onboarding matched to users’ readiness levels). Qualitative evidence reinforced core pedagogical hallmarks, clear objectives, logical sequencing, and engaging practice, delivered via tailored AI-driven learning paths that augment rather than replace sound instructional design. Instructor competency (β = 0.289) remains pivotal in AI-enhanced VR, encompassing fluency with AI systems, virtual-interaction dynamics, and the orchestration of human guidance with AI feedback. Perceived usefulness had a more negligible yet significant effect (β = 0.251); in highly immersive, well-designed contexts, absorption may emerge even when initial usefulness appraisals are modest, though multidimensional usefulness (real-time learning, long-term development, transfer, efficiency) still shapes willingness to engage deeply.

### Incremental value of AI enhancement

TEEL’s incremental validity over standard VR (ΔR² = 0.164) indicates a step change attributable to AI features, adaptive difficulty, personalized feedback, intelligent error correction, and predictive analytics, rather than a marginal improvement. Participants consistently linked these features to higher engagement and faster learning.

### Boundary conditions and group differences

Core relations were robust across demographics, but effects were more substantial among more experienced learners, suggesting that expertise facilitates integration of technological and pedagogical inputs and attention to content over interfaces. Older participants achieved comparable outcomes with additional early support, underscoring the importance of considering demographic factors when implementing AI-enhanced training.

### Design implications

Design recommendations from qualitative data include progressive, confidence-building onboarding; transparent progress tracking; immediate, relevant feedback; challenge-calibrated adaptivity; and seamless integration of AI and human instructional approaches, all oriented toward eliciting cognitive absorption. Features should be evaluated primarily by their capacity to evoke deep engagement, rather than solely by technical sophistication.

### Limitations and future directions

Limitations include the cross-sectional design (temporal causality requires a longitudinal study) and some self-report measures (mitigated through mixed methods). Future work should examine the temporal dynamics of absorption (to optimize session length/spacing), social presence, and collaborative learning in AI-VR, as well as objective indices of absorption (physiological signals, behavioral telemetry). This study presents an initial test of A-W theorizing in a rich martial arts context and motivates broader evaluations across various skill levels, educational backgrounds, and cultures.

## Conclusion

This research developed and validated the TEEL framework through a mixed-methods study involving 847 practitioners across six countries. SEM results showed all hypothesized links were statistically significant and directionally consistent: technology readiness (β = 0.387) was the strongest predictor of cognitive absorption, followed by instructional design quality (β = 0.312), instructor competency (β = 0.289), and perceived usefulness (β = 0.251). The association between cognitive absorption and learning effectiveness was strong (β = 0.791), indicating that complete mediation exists, where antecedents influence outcomes through cognitive absorption rather than directly. The model explained 73.2% of the variance in learning performance and 68.9% in cognitive absorption, and AI-enhancement components yielded significant incremental validity (ΔR² = 0.164) over standard VR. Collectively, the evidence supports prioritizing features that evoke cognitive absorption when designing AI-enhanced VR training systems and justifies the added complexity and cost of AI for next-generation immersive learning.

## Data Availability

The anonymized datasets generated and analyzed during the current study are available from the corresponding author upon reasonable request.
